# *Myracrodruon urundeuva* seed exudates proteome and anthelmintic activity against *Haemonchus contortus*

**DOI:** 10.1371/journal.pone.0200848

**Published:** 2018-07-19

**Authors:** Alexandra M. S. Soares, Jose T. A. Oliveira, Cláudia Q. Rocha, André T. S. Ferreira, Jonas Perales, Ana Caroline Zanatta, Wagner Vilegas, Carolina R. Silva, Livio M. Costa-Junior

**Affiliations:** 1 Laboratory of Plant Biochemistry, Chemical Engineering Program, Exact Sciences Center and Technology, Federal University of Maranhão, Sao Luís, Maranhão, Brazil; 2 Laboratory of Plant Defense Proteins, Federal University of Ceará, Campus do Pici, Fortaleza, Ceará, Brazil; 3 Laboratory of Advanced Studies in Phytomedicines, Department of Chemistry, Center for Exact Sciences and Technology, Federal University of Maranhão, São Luís, Maranhão, Brazil; 4 Laboratory of Toxinology, Oswaldo Cruz Foundation, Rio de Janeiro, Rio de Janeiro, Brazil; 5 Laboratory of Natural Products, Institute of Biosciences, Bioprospecting, Coastal Campus of São Vicente, São Vicente, São Paulo, Brazil; 6 Laboratory of Parasite Control, Department of Pathology, Center for Biological and Health Sciences, Federal University of Maranhão, São Luís, Maranhão, Brazil; Banaras Hindu University, INDIA

## Abstract

Seed exudates are plant-derived natural bioactive compounds consisting of a complex mixture of organic and inorganic molecules. Plant seed exudates have been poorly studied against parasite nematodes. This study was undertaken to identify proteins in the *Myracrodruon urundeuva* seed exudates and to assess the anthelmintic activity against *Haemonchus contortus*, an important parasite of small ruminants. *M*. *urundeuva* seed exudates (SEX) was obtained after immersion of seeds in sodium acetate buffer. SEX was fractionated with ammonium sulfate at 0–90% concentration to generate the ressuspended pellet (SEXF1) and the supernatant (SEXF2). SEX, SEXF1, and SEXF2 were exhaustively dialyzed against distilled water (cut-off: 12 kDa) and the protein contents determined. Mass spectrometry analyses of SEX, SEXF1, and SEXF2 were done to identify proteins and secondary metabolites. The seed exudates contained protease, protease inhibitor, peptidase, chitinase, and lipases as well as the low molecular weight secondary compounds ellagic acid and quercetin rhamnoside. SEX inhibited *H*. *contortus* larval development (LDA) (IC_50_ = 0.29 mg mL^-1^), but did not affect larval exsheathment (LEIA). On the other hand, although SEXF1 and SEXF2 inhibited *H*. *contortus* LEIA (IC_50_ = 1.04 and 0.93 mg mL^-1^, respectively), they showed even greater inhibition efficiency of *H*. *contortus* larval development (IC_50_ = 0.29 and 0.42 mg mL^-1^, respectively). To the best of our knowledge, this study is the first to show the anthelmintic activity of plant exudates against a gastrointestinal nematode. Moreover, it suggests the potential of exuded proteins as candidates to negatively interfere with *H*. *contortus* life cycle.

## Introduction

Amongst various helminth species that parasitizes sheep and goats, the gastrointestinal nematode *Haemonchus* contortus (Barber’s pole worm) represents one of the major constraints on these small ruminant productions in temperate and tropical regions of the world [1,2]. To aggravate the problem, the resistant nematode strains to synthetic anthelmintic drugs is spreading and medicinal plants with anthelmintic properties have been used in traditional medicine as an alternative to synthetic chemical products offers a ray of hope.

Some plant proteins have *in vitro* and *in vivo* anthelmintic activity on parasitic nematodes at different life stages. For instance, proteins from *Spigelia anthelmia* inhibited egg hatching, larval exsheathment, and larval migration of *H*. *contortus* [3]. Cysteine proteinases from *Carica papaya* displayed anthelmintic activity against *Protospirura muricola* [4] and *Trichuris trichiura*, as revealed by randomized controlled trials conducted in pigs [5]. Moreover, the lectins phytohemagglutinin E3L (PHA-E3L), wheat germ agglutinin (WGA), and concanavalin A (Con A) had anthelmintic activity as they effectively disrupted the feeding of the first stage larvae of the gastrointestinal nematodes *Teladorsagia circumcincta*, *H*. *contortus*, and *Trichostrongylus colubriformis* in infected sheep [6]. Therefore, plant compounds, like proteins, constitute potential alternatives for controlling parasites of veterinary importance [7,8].

Plant exudates consist of complex mixtures of organic and inorganic molecules, including carbohydrates, proteins, volatile compounds, and inorganic ions. Although studies on antimicrobial, anthelmintic, anti-inflammatory, antinociceptive, anti-ulcerogenic, antioxidant, and wound healing properties of plant exudates have been published [9], very few verified the activity of the seed exudates against nematodes. To the best of our knowledge, the anthelmintic potential of seed exudates against gastrointestinal nematodes was not previously reported.

Seed exudates protect seed during germination from soil pathogens [10]. For example, the seed exudates of *Cicer arietinum*, which contains chitinase, chitosanase, and protease inhibitors, has antifungal activity against *Fusarium oxysporum* [10,11]. Recently, Rocha et al. [12] reported that the *Glycine max* seed exudates contains plant-defense proteins active toward the nematode *Meloidogyne incognita*, a plant parasitic helminth. As chitin is a structural component of fungal cell wall [13] and eggshells of nematodes [14], bioactive compounds that degrade chitin may exert antifungal and anthelmintic effects.

*Myracrodruon urundeuva* (Portuguese common names: aroeira, aroeira-do-sertão [15], a tree belonging to the family Anarcadiacea, is highly exploited as a timber source, fuel, and medicinal in the semi-arid northeast region of Brazil. This plant has been noted for its antimicrobial, anti-inflammatory and analgesic properties, anti-leishmania, and larvicidal activity against *Aedes aegypti* [16–20]. Incidentally, none of these above properties were studied in seed exudates from *M*. *urundeuva*. Nevertheless, taken into consideration the anthelmintic potential of seed exudates compounds and interest in alternative control methods of gastrointestinal nematodes, this current study aimed to determine the seed exudates proteome of *M*. *urundeuva* and investigate its anthelmintic activity against *H*. *contortus*.

## Materials and methods

### Plant material

Mature seeds of *M*. *urundeuva*, free from chemical or physical treatments, were purchased from Arbocenter Seed Trade (Birigui, São Paulo, Brazil), under conformity number: 00050–15.

### *Myracrodruon urundeuva* seed exudates and derived fractions

Seeds were surface-sanitized with 30% (v/v) ethanol for 5 min. and rinsed exhaustively with distilled water. Three replicates of 40 seeds each were soaked in 12 mL 0.1 M sodium acetate buffer pH 5.0 at 5 °C and 24 later the exudates collected. After exhaustive dialysis against distilled water (*cut-off*: 12 kDa), the seed exudates (SEX) samples were collected and the volumes recorded.

Every SEX sample was saturated to 90% with ammonium sulphate and after standing for 12 h at 5 °C, they were centrifuged at 15,000 x *g*, 4°C for 30 min. The supernatants were recovered and the precipitates were ressuspended in distilled water. The precipitates (SEXF1) and supernatants (SEXF2) were exhaustively dialyzed against distilled water (cut-off: 12 kDa) and lyophilized. The protein contents were determined [21] using bovine serum albumin (BSA) as the standard protein.

### Proteomic analysis (LC-ESI-MS/MS)

Identification of proteins exuded from *M*. *urundeuva* seeds was performed according to Araújo et al. [16]. SEX, SEXF1, and SEXF2 samples (50 μg) were reduced with dithiothreitol and alkylated with iodoacetamide. Next, the samples were digested overnight with trypsin at a 1:50 (w/w) enzyme/substrate ratio. The tryptic peptides were analyzed using a reverse-phase (RP) column coupled to an LTQ-Orbitrap XL mass spectrometer on an nLC-Easy II system. The tryptic peptides samples were applied to the pre-equilibrated column with the mobile phase A [0.1% (v/v) formic acid in water]. Elution was carried out using the mobile phase B [0.1% (v/v) formic acid in acetonitrile] in a linear gradient mode of 2 to 40% acetonitrile. The peptides were subjected to collision-induced dissociation fragmentation and analyzed on a Linear Trap Quadrupole (LTQ) in MS/MS mode. The peptide mass profiles were evaluated using Peaks Studio. The LC-ESI-MS/MS data were processed and proteins were identified using the Uniprot viridiplantae database (searched entry: 3275215). The following default parameters were used: maximum of two missed cleavage by trypsin, fixed modification specified as carbamidomethylation (C), and oxidation (M) as variable modification; fragment mass error tolerance of 0.6 Da; the false discovery rate (FDR) values at protein levels was ≤1%, at least one unique peptide identified. Redundancy identified in the resulting list (proteins with the same values for -10lgp; coverage, #peptides; #unique, PTM; Avg Mass) was manually removed. Identified proteins common in the three studied samples (SEX, SEXF1, and SEXF2) were classified based on the Gene Ontology (GO) according to their biological process categories, using UniProtKB as the reference.

### Chromatography on HPLC and mass spectrometry analysis (FIA-ESI-IT-MS^n^)

SEX, SEXF1, and SEXF2 were individually solubilized in 2.0 mL methanol/water (9:1, v/v) and applied onto a C18 cartridge (Strata C18-E, Phenomenex). Eluted samples were filtered (Simplepure PTFE 0,22 μm, Allcrom) and diluted to around 5.0 mg mL^-1^ in methanol/water (8:2, v/v). Next, they were individually applied (10 μL) on a Luna 5 μm C18 100 A column (250 μm x 4.6 μm) coupled to a Shimadzu model HPLC system (Shimadzu Corp., Kyoto, Japan). Elution, at 1 mL min^-1^ flow rate and 20 °C, was carried out with solvent A (0.02% acetic acid in water) and B (0.02% acetic acid in methanol) using the following schedule: a 30 min. gradient from 95% A to 5% B, which was hold for 20 minutes. Data were collected and processed using the LC Solution software (Shimadzu).

For FIA-ESI-IT-MS^n^ analysis, direct flow infusion of the samples was performed on a Thermo Scientific LTQ XL linear ion trap analyzer equipped with an electrospray ionization (ESI) source, in negative mode (Thermo, San Jose, CA, USA). A stainless-steel capillary tube at 280 °C, 5.00 kV spray voltage, 90 V capillary voltage, 100 V tube lens, and 5 μL min^-1^ flow was used. Full scan analysis was recorded in m/z range from 100–1000. Multiple-stage fragmentations (ESI-MSn) were performed using the collision-induced dissociation (CID) method against helium for ion activation. The first event was a full-scan mass spectrum to acquire data on ions in that m/z range. The second scan event was an MS/MS experiment performed by using a data-dependent scan on the [M-H]- molecules from the compounds of interest at a collision energy of 30% and an activation time of 30 milliseconds. The product ions were submitted to further fragmentation under the same above conditions, until no more fragments were detected. Finally, the compounds were identified based on the fragments shown.

### Anthelmintic assays

*H*. *contortus* eggs were collected from sheep artificially infected according to Coles et al. [22]. Third-stage larvae (L3) were obtained from sheep infected with a monospecific strain of *H*. *contortus* [23, 24]. All animals were maintained indoors in individual pens and were fed a concentrated feed (68% corn, 26% wheat bran, 2% soybean, 2% calcareous and 2% mineral) representing 3% of the respective BW. Tifton grass hay (*Cynodon* sp.), water and mineral supplements specific for sheep were provided *ad libitum*. The experimental procedures were approved by the Animal Ethics Committee of the Federal University of Maranhão (protocol number 23115.005443/2017-51). Lyophilized SEX, SEXF1, and SEXF2 (10 mg) were ressuspended in phosphate buffer pH 7.2 [4.76 mM Na_2_HPO_4_; 1.76 mM KH_2_PO_4_; 137 mM NaCl; 2.7 mM KCl], and used in LDA (Larval development assay) and LEIA (Larval exsheathment inhibition assay).

LDA was carried out in 96-well culture plates [25], with modifications, in which 100 *H*. *contortus* eggs, suspended in 100 μL distilled water, were dropped in each well. After 24 h at 27 °C incubation in a B.O.D., the 1^st^ stage larvae (L1) were obtained. Next, 40 μL nutritive medium [*E*. *coli*, yeast extract and amphotericin B, from Sigma-Aldrich] were added followed immediately by addition of 110 μL SEX, SEXF1, or SEXF2 at 2.0, 1.0, 0.5, 0.25, and 0.0125 mg mL^-1^ final concentrations. Control samples consisted of L1, the nutritive medium, and phosphate buffer instead of the sample aliquots. Control and experimental samples were incubated at 27 °C for six days when the larval development was stopped by addition of Lugol’s iodine solution. L1 and L3 (3^rd^ stage larvae) in each well were counted using an inverted microscope [26].

LEIA was performed according to Jackson and Hoste [27] with modifications. SEX, SEXF1, and SEXF2 were individually dissolved in 1 mL phosphate buffer, pH 7.2, and added to viable *H*. *contortus* L3 (in 1 mL distilled water), at 1.2, 1.08, 0.972, 0.875, 0.787 mg mL^-1^ final concentrations, in quadruplicate from each sample. After incubation for 3 h, at 27 °C, and ≥80% RH, larvae from each well were washed with distilled water and centrifuged at 2,540 x *g*, at 24 °C. This washing process was twice repeated and larvae were immediately subjected to an artificial exsheathment process by contact with sodium hypochlorite (2,0%, w/v). For controls, samples were replaced by phosphate buffer, pH 7.2. Larval exsheathment was monitored after 0, 20, 40, and 60 min. incubation by microscopic observation at a 100× magnification and the percentage inhibition of exsheathed larvae were calculated.

### Statistical analysis

The experimental design used in all biological assays was completely randomized. The mean of each treatment was compared to its respective control. The data were initially transformed to Log(X), normalized and then nonlinear regression were calculated to get IC_50_ (50% inhibition concentration) for SEX, SEXF1, and SEXF2 using GraphPad Prism 7.0 software (GraphPad Inc., San Diego, CA, USA).

## Results

### Seed weigh and protein contents of seed exudates

**Fig 1:**
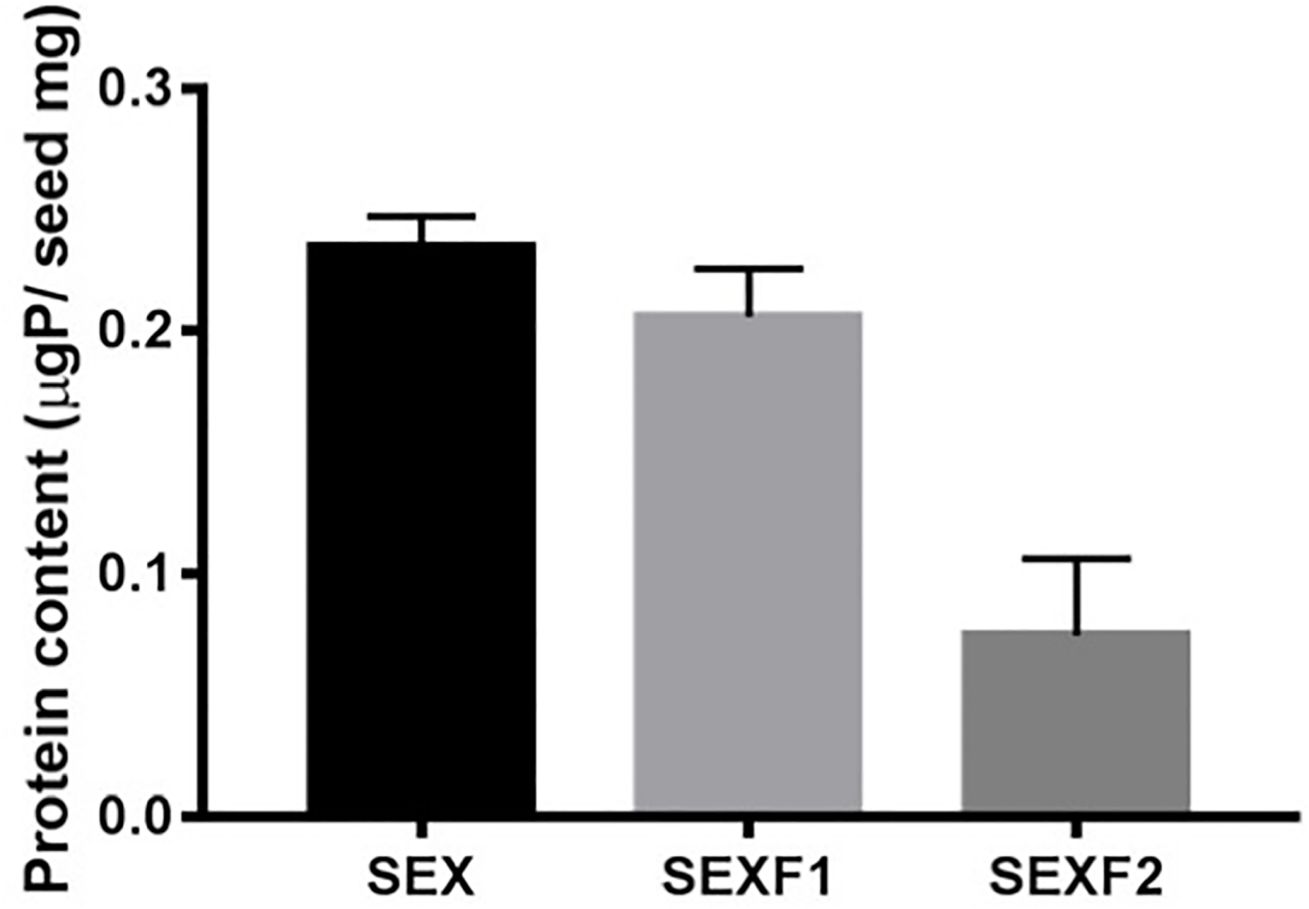
Protein content of seed exudate (SEX), ressuspended pellet (SEXF1) and the supernatant (SEXF2) of *Myracrodruon urundeuva*.

The mean of a *M*. *urundeuva* mature seed weight was 13.9 mg. SEX, SEXF1, and SEXF2 weighed an average of 0.24, 0.21, and 0.08 μg protein/mg seed, respectively (Fig 1).

### Identification of *Myracrodruon urundeuva* seed exudate proteins

Mass spectrometry analyses of the *M*. *urundeuva* seed exudates indentified 60, 89, and 175 proteins in SEX, SEXF1, and SEXF2, respectively [S1 Table]. Six common proteins were identified over the three samples: two heat shock proteins (A0A067DCX3, A0A061FNG5), actin (D9J011), two chitinases (D3TIC1, A0A067DS11), and monodehydroascorbate reductase (A0A022R646). Additionally, proteins previously reported to possess anthelmintic activities like protease (A0A067JBB7) and protease inhibitor (A0A067JPF4) were identified in SEXF1, whereas lipase (A0A1R3GK12) was identified in SEXF2.

### Identification of low molecular weight non-protein constituents in *Myracrodruon urundeuva* seed exudates

**Fig 2:**
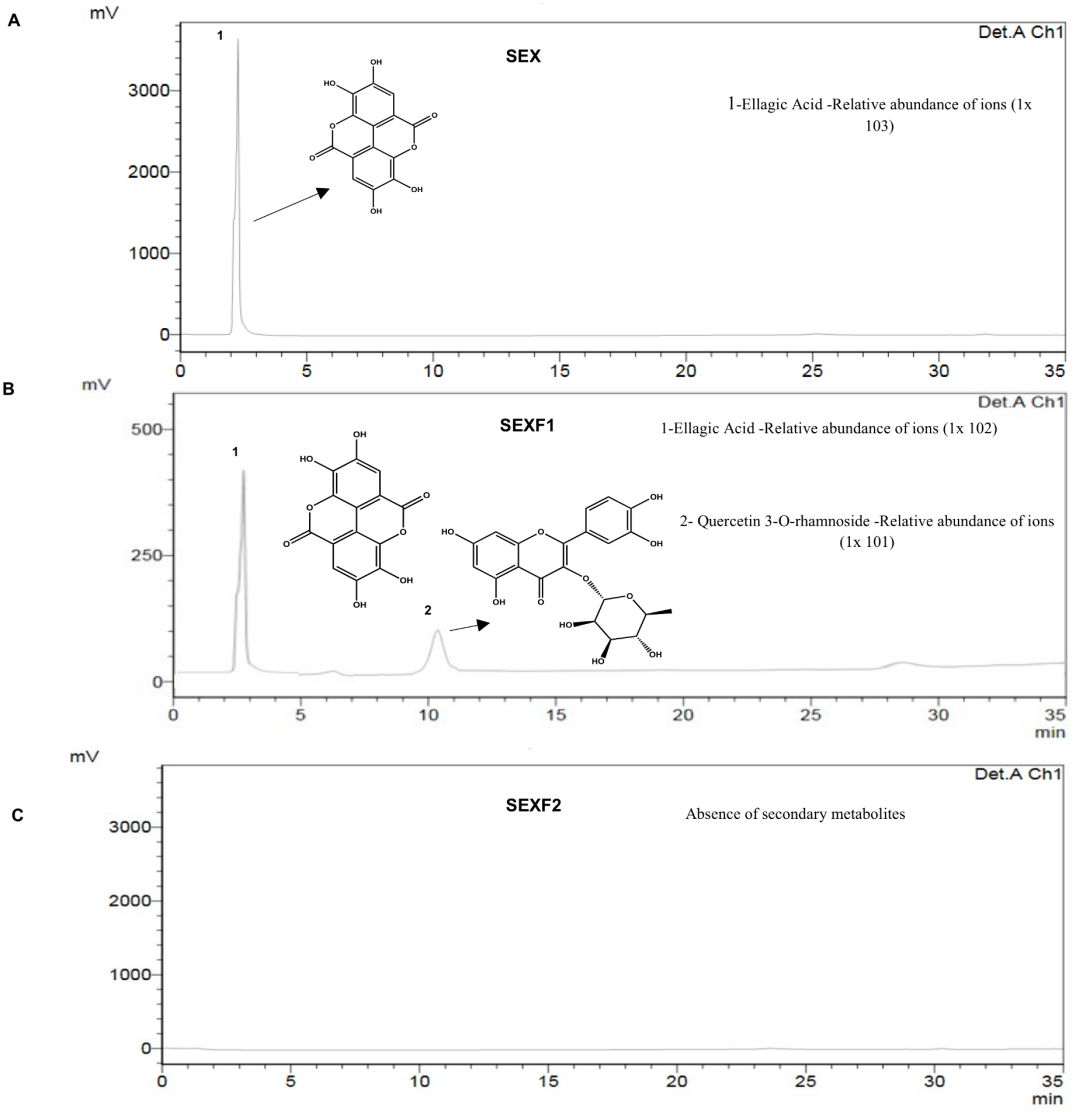
Chromatogram profiles and identification of low molecular weight non-protein constituents of seed exudate–SEX (A), ressuspended pellet—SEXF1 (B) and the supernatant—SEXF2 (C) of *Myracrodruon urundeuva* after HPLC and LC-ESI-IT/MS analyses.

With the aid of an ultraviolet detector (UV/Vis) coupled to an HPLC system, one peak was detected in SEX (3.6 x 10^2^ mV) and two peaks (2.85 x 10^2^ and 0.9 x 10^1^ mV, respectively) in SEXF1 (Fig 2). SEXF2 had no detected peaks.

### In vitro anthelmintic activity against *Haemonchus contortus*

SEX inhibits the larval development of *H*. *contortus* (IC_50_ 0.29 mg mL^-1^). SEXF1 and SEXF2 presented IC_50_ of 0.29 and 0.42 mg mL^-1^, respectively, on LDA. Although no larval exsheathment inhibition was verified in SEX treatment, under the experimental conditions used, SEXF1 and SEXF2 showed IC_50_ of 1.04 and 0.93 mg mL^-1^, respectively (Table 1, S1 Fig).

**Table 1:** Concentration (mg mL^-1^) of seed exudate (SEX), ressuspended pellet (SEXF1) and the supernatant (SEXF2) of *Myracrodruon urundeuva* required for achieving 50% inhibition (IC_50_) of *Haemonchus contortus* in larval development (LDA) and exsheathment (LEIA) at its 95% confidence interval.

Samples	IC_50_ mg mL^-1^ (95% CI)	
	LDA	LEIA
SEX	0.29 (0.231–0.353)^a^	> 1.2
SEXF1	0.29 (0.245–0.347)^a^	1.04 (0.994–1.081)^a^
SEXF2	0.42 (0.356–0.492)^b^	0.93 (0.913–0.945)^a^

LDA: larval development assay; LEIA: larval exsheathment inhibition assay. 95% CI = 95% confidence interval. Different letters represent significant differences between treatments (P>0.05).

## Discussion

The amount of proteins of 0.24 μgP/mg seed (3.42 ±0.29 μgP/seed) exudated from *M*. *urundeuva* seeds after soaking in 0.1 M sodium acetate buffer, pH 5.0, at 5 °C, for 24 h, followed by dialysis (cutoff: 12 kDa) against distilled water, was very low compared with that obtained for soybean seeds soaking for 18 h in 0.1 M sodium acetate buffer, pH 5.0, at 28 °C, and dialyzed (cutoff: 3.5 kDa) against distilled [12]. Besides *M*. *urundeuva* and soybean seeds belong to different plant species, the lower temperature used in the present work to avoid microbial proliferation, soaking duration, and the dialysis tubing separation determined by the molecular weight-cutoff of the dialysis membranes might have implications. For example, in our case, we only measured the content of proteins with molecular weight higher than 12 kDa, whereas Rocha et al. [12] also evaluated peptides. Nevertheless, it is known that the seed exudates composition varies with the plant species, soil (pH, composition), temperature, and the presence of microorganisms [11].

As expected, most proteins were concentrated in SEXF1 (87% of the total proteins) in relation to SEXF2 (Fig 1). After LC-ESI-MS/MS analyses of the studied samples, 60, 89, and 175 proteins were identified in SEX, SEXF1, and SEXF2, respectively (S1 Table). Low abundance of proteins can be enriched from crude extracts by precipitation methods [28]. Thus, ammonium sulfate protein fractionation was of utmost importance to allow identification of proteins present in the *M*. *urundeuva* seed exudates.

The common proteins identified in SEX, SEXF1, and SEXF2 are involved in different biological processes. Monodehydroascorbate reductases are known to play protective roles in plants. For instance, they are increased in *Malpighia glabra* leaves under cold and salt stress conditions [29]. Actin is a structural constituent of cell cytoskeleton, involved in development, reproduction, and cellular responses to biotic and abiotic stresses, involved in physical barrier formation and transport of antimicrobial compounds to the site of infection in plants [30]. Chitinase is involved in carbohydrate metabolic process, cell wall macromolecule catabolic process, and chitin catabolic process. Chitinases increased in abundance in response to a variety of stress conditions being involved in plant responses to cold, for example [31, 32]. Lipases catalyze the hydrolysis of carboxylic acid ester bonds, playing a central role in the release of fatty acids from the reserve triacylglycerols in the germinating seeds. Additionally, seed lipases can quickly hydrolyze a great variety of fatty acids [33, 34]. Heat shock proteins are related to development, seed germination, as well as to defense response. For instance, the expression of heat-shock proteins can be induced by salinity, cold or heat [35]. Proteases catalyze the hydrolysis of proteins in a process known as proteolysis [36]. In addition to several other functions, plant proteases have been implicated in remobilization of seed protein reserves during germination [37] and can be exuded to degrade proteins in surrounding rhizosphere to allow amino acid nutrition by soil microorganisms [38].

Scarafoni et al. [39] verified that the proteome profile of the *Lupinus albus* seed exudates varies during the time of exudation, with discharge of pre-formed proteins over the first 12 h and after that, increased protein release and drastic change in composition. The authors suggested that the released proteins may protect the spermosphere environment acting as the first defense against pathogens.

The studied samples were exhaustively dialyzed (cut-off: 12 kDa) against distilled water and non-protein low molecular weight compounds should not be present. Ellagic acid was detected both in SEX and SEXF1, although at low amounts. Additionally, in SEXF1, a quercetin-derived compound was also found (Fig 2). Probably, this compound was not detected in SEX, from which SEXF1 is derived, because of its relative abundance, below to the detection level. because the ion suppression reducing detector response, or signal: noise as a manifested effect of competition for ionisation efficiency in the ionisation source, between the analyte(s) of interest and other endogenous or exogenous [40]. However, we believe that ellagic acid and quercetin rhamnoside were somehow linked with proteins and were not excluded by dialysis. When solvents were added to conduct HPLC and FIA-ESI-IT-MS^n^ experiments, the compounds were detached from the proteins and were detected. Although ellagic acid and quercetin previously showed anthelmintic properties against *H*. *contortus* [41–43], no correlations can be done with the presence of these low molecular weight compounds with the anthelmintic properties of the exudates because ellagic acid and quercetin rhamnoside were not detected in SEXF2, which is effective towards *H*. *contortus* (Table 1).

The *M urundeuva* seed exudates and derived protein fractions display anthelmintic activity, mainly on *H*. *contortus* LDA (Table 1). Compared to SEX, this inhibitory effect did not change in SEXF1, but in SEXF2 it was lower (Table 1). Such result suggests that the compound(s) particularly present in SEXF1, which is a protein-enriched fraction, promoted inhibition of *H*. *contortus* larval development. On the other hand, inhibition of *H*. *contortus* larval exsheathment (LEIA) was equally distributed between SEXF1 and SEXF2 suggesting that both fractions have compounds with LEIA activity. Ammonium sulfate precipitation of SEX did not enhance the LC_50_ value for SEXF1 neither for SEXF2. Considering the hypothesis that exuded proteins are responsible for such activity, it is possible that some of them have been denatured and/or have aggregated to each other during precipitation, resuspension, dialysis against water, and lyophilization process, followed by resuspension to carry out the tests, weakening the inhibition effect on *H*. *contortus* LDA. Other hypothesis that can be put forward is that SEXF2 might have compounds that exert a synergistic effect when mix to SEXF1 like they occur naturally in SEX. Nevertheless, further studies are needed to purify the active principle present in *M urundeuva* seed exudates towards its biochemical and biological characterization.

Diverse proteins have been studied for nematicidal activity [3, 44, 45]. The proteomic profiles of SEX, SEXF1, and SEXF2 revealed the presence of proteins that potentially can affect the nematode development cycle. Proteases can weaken the nematode cuticle structure by enzymatic degradation of constituent proteins, which can lead to parasite disintegration due to the internal pressure level in the nematode body cavity [4, 46]. Protease inhibitors have potential for controlling *H*. *contortus* development because the importance of proteases in this process [47, 48]. Chitinase can deplete fibrillar components of the medial region of *H*. *contortus* eggshell [49]. Lipases have also potential anthelmintic compounds, as lipids play important roles in the lipid metabolism of nematodes [50] and have structural role in *H*. *contortus* cuticle. Therefore, as the eggshell of *H*. *contortus* presents an outer vitelline layer, chitin in the medial region, and a basal layer composed by lipid and protein [49], and the nematode cuticle contains proteins, exposure of *H*. *contortus* eggs and larvae to the *M*. *urundeuva* seed exuded proteases, protease inhibitors, chitinases, and lipases could interfere with the normal life cycle of the parasite, for example.

In the present study, we show for the first time the proteome profile and the potential of proteases, proteases inhibitors, chitinases, and lipases exuded by *M*. *urundeuva* seeds to negatively interfere with *H*. *contortus* life cycle. However, further studies must be conducted to clearly elucidate the effective anthelmintic compound(s).

## Supporting information

S1 Fig Larval exsheathment of *Haemonchus contortus* in the presence of ressuspended pellet (SEXF1) and the supernatant (SEXF2) of *Myracrodruon urundeuva*. (DOCX)

S1 Table *Myracrodruon urundeuva* seed proteins identified by LC-ESI-MS/MS. (XLSX)
