# Supplementary material for: Myracrodruon urundeuva seed exudates proteome and anthelmintic activity against Haemonchus contortus
Source: PLoS One. 2018 Jul 19;13(7):e0200848. doi: 10.1371/journal.pone.0200848 (PMC6053183; doi:10.1371/journal.pone.0200848)
Supplement: Supplementary file 1 [file pone.0200848.s001.docx]

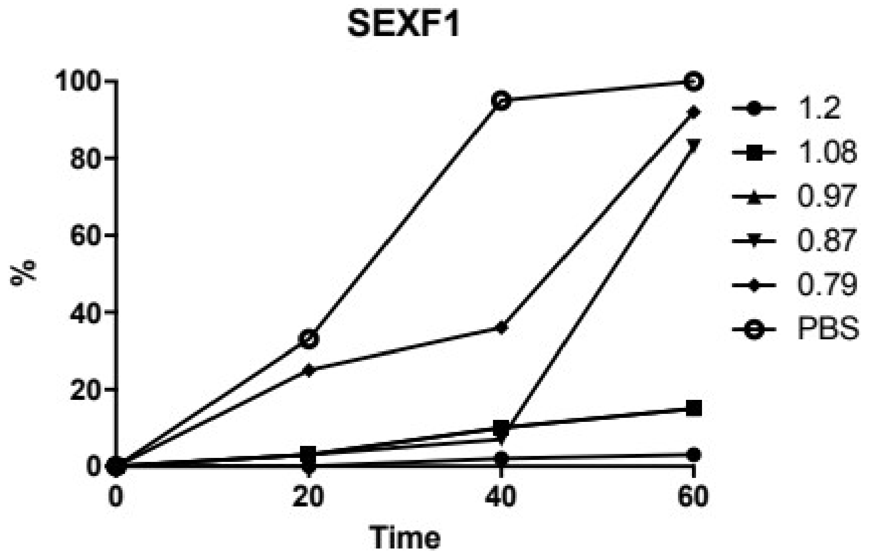
SEXF1
100
80
60
40
20
0
%
0
20
40
60
Time
1.2
1.08
0.97
0.87
0.79
PBS

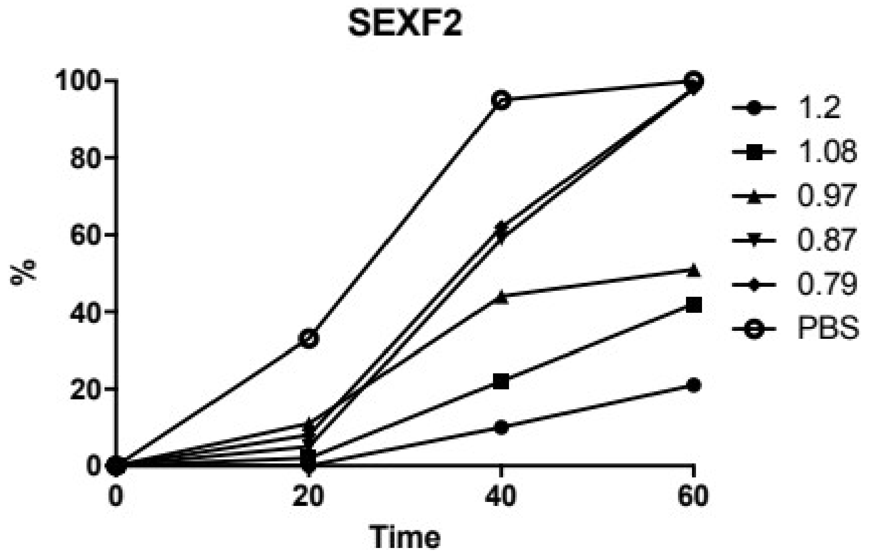
SEXF2
100
80
60
40
20
0
%
0
20
40
60
Time
1.2
1.08
0.97
0.87
0.79
PBS
